# Molecular Understanding of the Activation of CB1 and Blockade of TRPV1 Receptors: Implications for Novel Treatment Strategies in Osteoarthritis

**DOI:** 10.3390/ijms19020342

**Published:** 2018-01-24

**Authors:** Jakub Mlost, Magdalena Kostrzewa, Natalia Malek, Katarzyna Starowicz

**Affiliations:** 1 Laboratory of Pain Pathophysiology, Department of Pain Pharmacology, Institute of Pharmacology Polish Academy of Sciences, 31-343 Krakow, Poland; mlost13@gmail.com (J.M.); m.kostrzewka@gmail.com (M.K.); en.malek@gmail.com (N.M.); 2 Department of Neurochemistry, Institute of Pharmacology Polish Academy of Sciences, 31-343 Krakow, Poland

**Keywords:** osteoarthritis, endocannabinoids, OMDM198, TRPV1, sensitization

## Abstract

Osteoarthritis (OA) is a joint disease in which cartilage degenerates as a result of mechanical and biochemical changes. The main OA symptom is chronic pain involving both peripheral and central mechanisms of nociceptive processing. Our previous studies have implicated the benefits of dual- over single-acting compounds interacting with the endocannabinoid system (ECS) in OA treatment. In the present study, we focused on the specific molecular alterations associated with pharmacological treatment. OA was induced in Wistar rats by intra-articular injection of 3 mg of monoiodoacetate (MIA). Single target compounds (URB597, an FAAH inhibitor, and SB366791, a TRPV1 antagonist) and a dual-acting compound OMDM198 (FAAH inhibitor/TRPV1 antagonist) were used in the present study. At day 21 post-MIA injection, rats were sacrificed 1 h after i.p. treatment, and changes in mRNA expression were evaluated in the lumbar spinal cord by RT-qPCR. Following MIA administration, we observed 2-4-fold increase in mRNA expression of targeted receptors (*Cnr1*, *Cnr2*, and *Trpv1*), endocannabinoid degradation enzymes (*Faah*, *Ptgs2*, and *Alox12*), and TRPV1 sensitizing kinases (*Mapk3*, *Mapk14*, *Prkcg*, and *Prkaca*). OMDM198 treatment reversed some of the MIA effects on the spinal cord towards intact levels (*Alox12*, *Mapk14*, and *Prkcg*). Apparent regulation of ECS and TRPV1 in response to pharmacological intervention is a strong justification for novel ECS-based multi-target drug treatment in OA.

## 1. Introduction

Osteoarthritis (OA) is a chronic joint disease in which cartilage degenerates as a result of its mechanical and biochemical changes followed by a low-grade inflammatory response [1]. On the molecular level, OA is characterized by collagen matrix disorganization and a decrease in proteoglycan content within the cartilage. Without the protective effects of the proteoglycans, the collagen fibres of the joint tissue become susceptible to degradation and thus exacerbate this process. Breakdown products when released into the extracellular matrix can evoke an inflammatory response in the synovium and therefore lead to a painful sensation on the peripheral level [2]. The hallmark symptom of osteoarthritis is pain, which is a mixed phenomenon involving nociceptive and neuropathic mechanisms at both the peripheral and central levels [3]. Indeed, patients with OA often develop allodynia and have lowered pain thresholds at locations distant from the place of the injury, suggesting an additional neuropathic mechanism [4].

Unfortunately, the current understanding of OA pathophysiological mechanisms has not led to the development of disease-modifying drugs that are able to stop or slow down disease progression. Present-day treatment is mostly based on palliative care, using nonsteroidal anti-inflammatory drugs (NSAIDs) such as ibuprofen, naproxen or diclofenac. However, they do not always provide adequate pain relief and may be associated with the onset of central sensitization during OA development [4]. More importantly, the use of NSAIDs is limited because they exert serious side effects on the gastrointestinal system and have cardiovascular effects, especially with prolonged use, including bleeding, ulcers, stroke, and myocardial infarction [5].

Compelling evidence suggests an active participation of the ECS in the pathophysiology of joint pain associated with OA. Cannabinoid receptor type 1 (CB1) agonists were proven to be effective analgesics in various animal models of chronic pain, including OA [6,7,8,9]. However, they are similar to NSAIDs and their clinical potential is highly limited by central nervous system-related side effects such as dizziness, memory impairment, euphoria and risk of abuse or addiction [10]. Alternative treatment strategies aimed at ECS stimulation have focused on inhibition of endocannabinoid degradation. This strategy brings various advantages over direct CB1 activation. Increase in endogenous endocannabinoid tone is devoid of psychoactive effects and will probably minimize side effects observed with systemic CB1 activation. Indeed, preclinical research has confirmed the antinociceptive potential of increased endocannabinoid levels together with an absence of cannabimetic effects [11] and has demonstrated that endocannabinoids adaptively dampen nociceptive transmission in OA [12].

Unfortunately, the inhibition of endocannabinoid degradation did not produce satisfactory results in the clinics. This may be due to redundancy in the metabolic pathways of ECS [13] and/or the activity of endocannabinoids at receptors distinct from the cannabinoid receptors [14]. For example, anadamide (AEA) and some of its lipoxygenation products are also highly potent ligands for the transient receptor potential vanilloid 1 (TRPV1) receptor, suggesting an interplay between ECS and the endovanilloid system. TRPV1 co-localizes with CB1 [15,16,17] and CB2 [18] in nervous tissue. This colocalization may influence pain transmission pathways through intracellular mechanisms. For example, the stimulation of CB1 can either inhibit or potentiate TRPV1 stimulation by its ligands, depending on whether or not the cyclic adenosine monophosphate (cAMP) signalling pathway is concomitantly activated [19]. Studies have shown that TRPV1 sensitivity can be enhanced by phosphorylation through adenylate cyclase and cAMP-dependent protein kinase A (PKA) [20]. It is possible that during inflammation, enhanced cAMP levels lead to TRPV1 phosphorylation through PKA and that CB1 counteracts the sensitization by inhibition of adenylate cyclase [21,22,23]. In fact, our previous research revealed upregulation of TRPV1 sensitizing factors, mitogen-activated protein kinase (MAPK), PKA and protein kinase C (PKC) in a neuropathic pain model [24]. TRPV1 is also sensitized by proinflammatory factors such as 12- and 15-hydroperoxyeicosatetraenoic acid (12- and 15-HPETE), 12- and 15-hydroxyeicosatetraenoic acid (12- and 15-HETE) and other arachidonic acid metabolites [25]. This means that upon tissue damage and the consequent inflammation, sensitivity of receptors to noxious stimuli increases. Moreover, changes in extracellular environmental pH impact the neural cell excitability. In acidic conditions, AEA preferentially binds to TRPV1 instead of CB1, leading to membrane depolarization [26].

All this aforementioned evidence supports the hypothesis that in circumstances of Fatty acid amide hydrolase (FAAH) inhibition, the actions of TRPV1 may be enhanced and serve as a contributing factor to the lack of antinociceptive potential of FAAH inhibitors in the clinic. This effect could be omitted by simultaneously combining FAAH inhibition and TRPV1 antagonism. In fact, this strategy was already tested in our laboratory, and it was proven to be effective. Małek et al. demonstrated TRPV1 and FAAH colocalization in dorsal root ganglions (DRGs) and subsequently used OMDM198, a dual FAAH/TRPV1 blocker, in a battery of behavioural experiments on animals with MIA-induced OA. OMDM198 increased the pain threshold in pressure application measurement and restored impaired weigh bearing [27]. The current state of knowledge regarding ECS and endovanilloid interactions highly support the use of dual-target FAAH and TRPV1 blockers on both theoretical and behavioural levels. However, little is known about the actual molecular underpinnings of such compounds. The spinal cord is the first central site of sensory processing. The signal transmissions related to pain can be modified by facilitatory and inhibitory interactions in pain pathways. In fact, molecular changes on the spinal cord level contribute to the development of central sensitization phenomenon, resulting in chronic pain [28]. Thus, quantitative evaluation of ECS components in the spinal cord, after OMDM198 stimulation, is the subject of the current study.

## 2. Results

### 2.1. Changes in Cnr1, Cnr2, Faah, and Trpv1 Gene Expression in the Lumbar Spinal Cord

**Figure 1:**
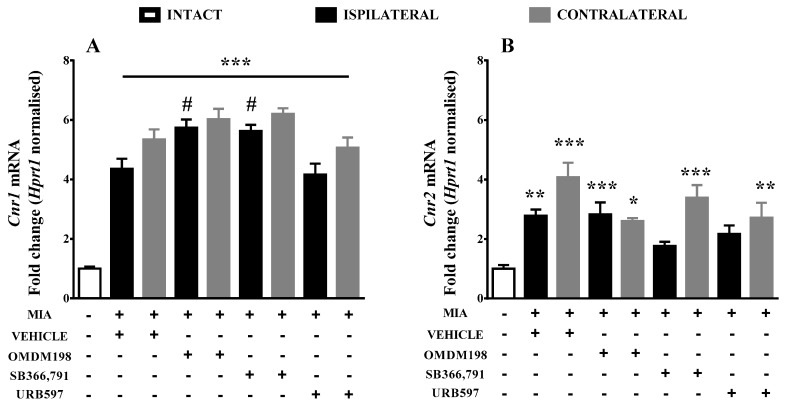

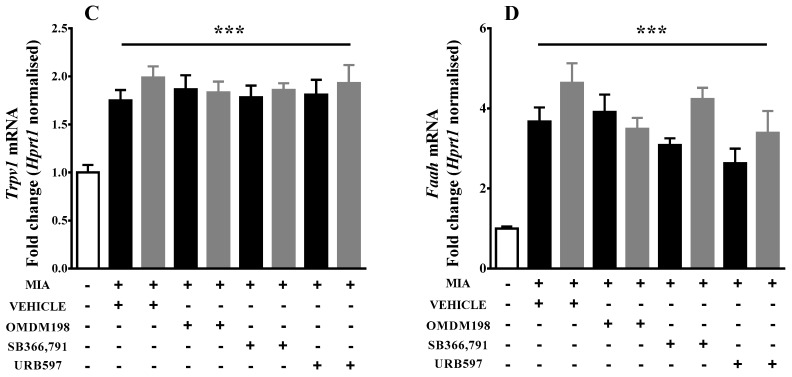
Results of qPCR analysis of *Cnr1* (**A**); *Cnr2* (**B**); *Trpv1* (**C**); and *Faah* (**D**); gene expression levels in the dorsal part of the lumbar spinal cord after MIA injection and treatment with OMDM198, SB366791 or URB597. Samples were collected 21 days after MIA injection, 1 h after i.p. administration of OMDM198, SB366791, URB597 or vehicle. Data are presented as the mean ± SEM and represent normalized averages derived from 6–10 samples for each group. The results are presented as a fold change normalized to the expression of the reference gene *Hprt1* compared to the intact animals. Statistical analysis was performed using one-way ANOVA followed by Bonferroni post hoc tests. Values with *p* < 0.05 were considered significant. * Denotes significant differences vs. intact; # denotes significant differences vs. the same side of the vehicle-treated osteoarthritic animals. **, ***, **** denote higher significance levels.

Gene expression analysis was performed in order to characterise changes in ECS components in OA and their response to pharmacological treatment. MIA injection caused significant upregulation of *Cnr1* (Figure 1A), *Cnr2* (Figure 1B), *Trpv1* (Figure 1C) and *Faah* (Figure 1D) expression in the lumbar spinal cord. Treatment with OMDM198 and SB366791 further increased *Cnr1* (CB1) expression exclusively on the ipsilateral side (6-fold change vs. 4-fold change, when compared to intact animals), whereas URB597 treatment did not affect levels of *Cnr1* expression in MIA-treated animals (Figure 1A). Treatment with OMDM198 had no influence on the *Cnr2* (CB2) elevated levels; however, SB366791 and URB597 abolished *Cnr2* upregulation from 2.8-fold change to 1.8- and 2.2-fold change, respectively on the ipsilateral side (Figure 1B). Furthermore, treatment either with OMDM198, SB366791 or URB597 did not change *Trpv1* and *Faah* expression levels (Figure 1C,D).

### 2.2. Changes in Gene Expression of Kinases Involved in TRPV1 Sensitization in the Lumbar Spinal Cord

**Figure 2:**
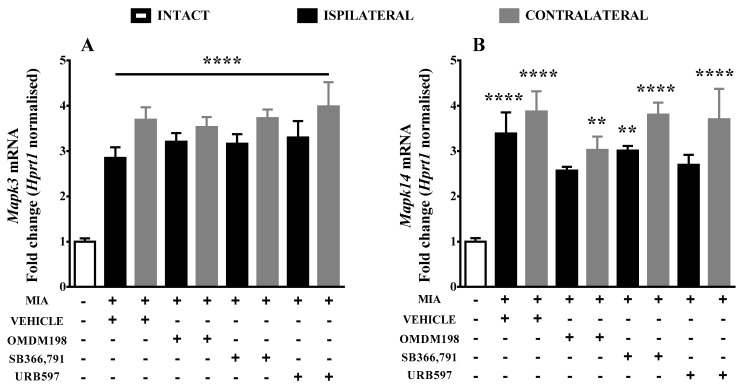

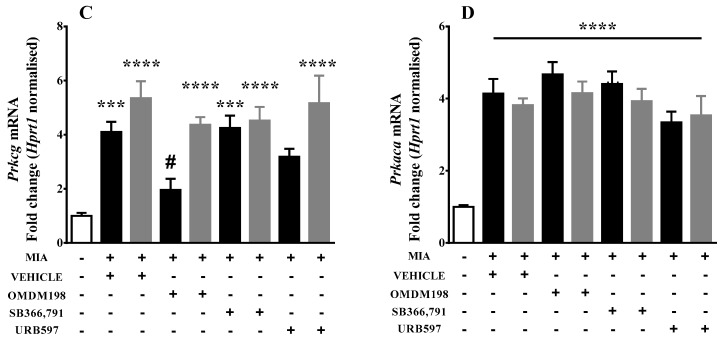
Results of qPCR analysis of *Mapk3* (**A**); *Mapk14* (**B**); *Prkcg* (**C**); and *Prkaca* (**D**) gene expression levels in the lumbar spinal cord after MIA injection and treatment with OMDM198, SB366791 or URB597. Samples were collected 21 days after MIA injection, 1 h after i.p. administration of OMDM198, SB366791, URB597 or vehicle. Data are presented as the mean ± SEM and represent normalized averages derived from 6–10 samples for each group. The results are presented as a fold change normalized to the expression of a reference gene *Hprt1* compared to the intact animals. Statistical analysis was performed using one-way ANOVA followed by Bonferroni post hoc tests. Values with *p* < 0.05 were considered significant. * Denotes significant differences vs. intact; # denotes significant differences vs. the same side of the vehicle-treated osteoarthritic animals. **, ***, **** denote higher significance levels.

For better understating of the molecular mechanisms of OMDM198 superior analgesic potential in the MIA model, we performed experiments aimed at measuring gene expression of several kinases involved in TRPV1 receptor sensitization. The analysis was performed at the lumbar spinal cord level, where sensitization of pain pathways occurs. Our results revealed strong upregulation (around 3–5-fold change) of *Mapk3*, *Mapk14*, *Prkcg*, and *Prkaca* mRNA expression in the vehicle-treated osteoarthritic animals (Figure 2A–D). Moreover, OMDM198 treatment abolished *Mapk14* and *Prkcg* upregulation (drop from 3.4- and 4-fold change to 2.6- and 2-fold change, respectively, when compared to intact animals) in the ipsilateral side of the spinal cord (Figure 1B,C).

### 2.3. Changes of the AEA Alternative Degrading Enzymes Gene Expression in the Lumbar Spinal Cord

**Figure 3:**
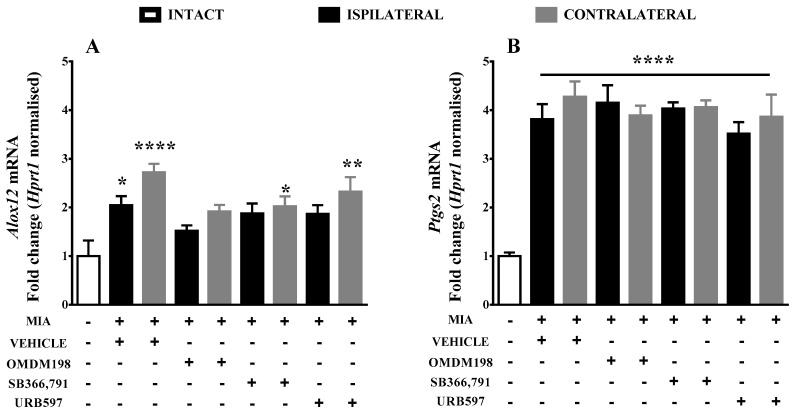
Results of qPCR analysis of *Alox12* (**A**) and *Ptgs2* (**B**) gene expression levels in the lumbar spinal cord after MIA injection and treatment with OMDM198, SB366791 or URB597. Samples were collected 21 days after MIA injection, 1 h after i.p. administration of OMDM198, SB366791, URB597 or vehicle. Data are presented as the mean ± SEM and represent normalized averages derived from 6–10 samples for each group. The results are presented as a fold change normalized to the expression of a reference gene *Hprt1* compared with the intact animals. Statistical analysis was performed using one-way ANOVA followed by Bonferroni post hoc tests. Values with *p* < 0.05 were considered significant. * Denotes significant differences vs. intact. **, ***, **** denote higher significance levels.

Finally, we decided to look further at enzymes involved in alternative degradation pathways of AEA for better understanding of FAAH inhibition consequences in OA. Osteoarthritic pain led to an upregulation of mRNA encoding enzymes of anandamide alternative synthesis pathways in the lumbar spinal cord (Figure 3). The abundance of *Alox12* mRNA was significantly increased on the ipsi- and contralateral side in MIA animals treated with vehicle (Figure 3A), 2-fold and 2.7-fold increase respectively. The *Alox12* upregulation was reduced by half following OMDM198 administration. Statistically significant changes were also observed on the contralateral side after SB366791 administration (2-fold increase) and after URB597 treatment (2.3-fold increase) (Figure 3A). *Ptgs2* (COX2) transcript levels increased 4-fold in OA rats compared with the intact animals (Figure 3B), and none of the tested compounds had an influence on it. Authors own results showed no significant changes in the expression of *Alox15* in the lumbar spinal cord at day 21 during OA development. We did not elucidate further pharmacological effects of this gene.

## 3. Discussion

OA is a debilitating disease with an underlying pathophysiology that is complex and not entirely understood. Joint degeneration seems to be a result of mixed environmental and genetic features. Due to the lack of self-healing capacity of articular cartilage, OA is among the most challenging joint diseases. The goal of OA research is to search for new therapeutic strategies that could prevent, reduce or stop the progression of the disease or, alternatively, resolve the existing damage to the joint. Studies have driven the identification of promising targets for OA treatment involving inflammation and/or nerve sensitization mechanisms in OA development, rationalizing our focus on the role of ECS as useful for the treatment of OA. Here, we have presented results describing molecular changes in ECS components in a MIA model of OA and provide novel insights regarding the pharmacological effects of endocannabinoids on OA considering ECS redundancy.

First, our qPCR analysis revealed strong mRNA upregulation in all the studied ECS components (*Cnr1*, *Cnr2*, *Faah* and *Trpv1*) on both sides of the lumbar spinal cord in response to OA induction (MIA treatment). That implies strong activation of ascending pain pathways in the spinal cord and a significant role of ECS in pain transmission. This observation is consistent with previous experiments revealing increased ECS tone as a pathophysiological mechanism involved in silencing sensitized pain pathways due to inflammation or nerve injury [29,30]. Our recent study by Małek et al. revealed that OMDM198 exerts more pronounced analgesia than single target drugs and this effect is blocked by a CB1 antagonist (AM251) [27]. In the present study, further increases in *Cnr1* expression were observed following TRPV1 blockade with both multi- and single-target drugs. CB1 upregulation may lead to a significant increase in efficacy and an analgesic potential of partial agonists [31], such as AEA, and, therefore, may provide an explanation for pronounced antinociceptive effects of OMDM198 in comparison to the single target FAAH inhibitor, URB597. These results hold a good promise for further research upon ECS as a potential treatment target in OA, because ECS stimulation could prevent or reduce this promiscuous form of pain plasticity. Additionally, the observed increase in *Trpv1* expression in the spinal cord confirms its role in the sensitization of pain pathways [32] and represents a suitable indicator for the use of dual-target drugs for OA treatment.

Increases in mRNA expression of kinases involved in TRPV1 sensitization indicates undergoing plasticity in pain pathways on the spinal cord level. This result is consistent with previous results obtained in our laboratory that revealed increased expression of *Mapk14*, *Prkcg*, and *Prkaca* in the peripheral nervous system in a chronic constriction injury model of neuropathic pain [24]. What is even more interesting is the fact that FAAH inhibition by OMDM198 or URB597 partially counteracted *Mapk14* and *Prkcg* upregulation in the ipsilateral side of the spinal cord and might represent their molecular mechanism of action. Ji et al. have shown that intrathecal administration of SB203580, a p38-mitogen-activated protein kinase (encoded by the *Mapk14* gene) inhibitor, decreases inflammatory heat hyperalgesia and induces TRPV1 protein upregulation in the DRGs [33,34]. Furthermore, recent research revealed that H89, a PKA (encoded by the *Prkaca* gene) inhibitor and GF109203X, a PKCγ (encoded by the *Prkcg* gene) inhibitor suppresses TRPV1 externalization in DRGs sensory neuron cultures [35] and that the latter compound is also able to reverse capsaicin-induced pain-related behaviour in MIA rats [36]. Decreases of *Mapk14* and *Prkcg* mRNA levels following FAAH inhibition highlights hopes for new treatment strategies of neuropathic components in rheumatic diseases, as seen with intrathecal administration of p38α MAPK and PKCγ inhibitors in a chronic constriction injury model of neuropathic pain [37,38].

An interesting finding of this work is a closer look at the alternative degrading pathways of AEA that could reveal some new insight into endocannabinoid metabolism under pathological conditions and point to beneficial analgesic effects in a therapeutic context. The *Alox12* gene is coding lipooxygenase-12, an enzyme involved in the transformation of arachidonate compounds such as arachidonic acid (AEA precursor) or AEA into proinflammatory factors such as 12-HETE (TRPV1 agonist). Previously mentioned research by Kelly et al. has shown elevated levels of 12-HETE in the knee joint and TRPV1 in the spinal cord of MIA rats [39]. Moreover, these studies confirmed a functional role of TRPV1 in a lowered mechanical threshold of knee joint afferents together with the analgesic potential of a TRPV1 blockade [39]. In the present study on a MIA model of OA, we observed a bilateral increase in *Alox12* expression in the spinal cord. These data add up to the relevance of the endovanilloid system on the spinal level of pain transmission in OA pathophysiology and further indicate the usefulness of TRPV1 antagonists as analgesic agents. Additionally, we observed bilateral attenuation of *Alox12* upregulation following OMDM198 treatment but not after treatment with any of the single-target drugs, which acted only partially.

Another enzyme capable of AEA degradation is COX2 encoded by the *Ptgs2* gene. COX2 is induced in inflammatory conditions and the main metabolic pathway involves arachidonic acid transformation into proinflammatory prostaglandin 2 (PGE2), which is another factor promoting TRPV1 upregulation [35]. AEA may undergo direct oxygenation by COX2, resulting in the formation of prostamide F2α (PGF2α) [40], a mediator of inflammation and pain [41]. It was shown that PGF2α metabolite levels are increased in OA patients’ serum and synovial fluid [42]. PGF2α exerts profibrotic action by induction of collagen production that can further limit joint movement [43]. On the other hand, COX2 inhibition reversed spinal neuron hyperexcitability during inflammation [44] and produced endocannabinoid-mediated spinal antinociception in a CB1-dependent manner [45]. Our experiments demonstrated an increase in *Ptgs2* expression on the spinal level. Unfortunately, its expression was not altered by any of the tested drugs. It may be worthwhile to study further a combined administration of OMDM198 with COX2 inhibitors or dual inhibition of FAAH and COX2 as an even more complex treatment strategy for osteoarthritis. Indeed, a recent work by Palermo et al. addressed the synthesis and the potential anti-inflammatory use of FAAH and COX-2 multi-target ligand, ARN2508 [46].

**Figure 4:**
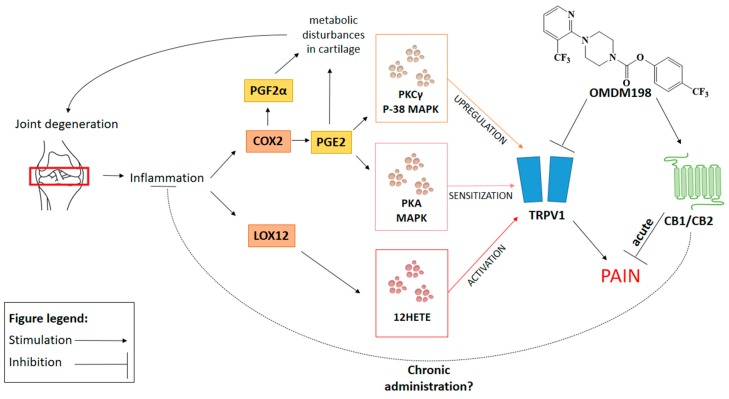
Schematic representation of the hypothetical molecular mechanism underlying OMDM198’s therapeutic potential in OA treatment.

To sum up, OMDM198 seems to exert its main effects through the antinociceptive profile of FAAH inhibition (indirect CB1 activation) and TRPV1 antagonism [27] but beyond that lies a complicated network of intercellular interactions. A summary of the proposed mechanisms of OMDM198 action is presented in Figure 4. Recent evidence pinpoints the role of inflammation in OA pathophysiology (reviewed in de Lange-Brokaar et al. 2012) [47], and the hereby presented results further identify its role in OA pathology. Our data recognized an increase in *Ptgs2* mRNA and its downstream effectors such as *Prkaca*, *Prkcg* and *Mapk14* [35], which could result in the observed TRPV1 upregulation, suggesting spinal sensitization as an important contributor to the development and progression of a neuropathic component of OA. These results are consistent with recent reports from two independent research groups [35,36] who neatly demonstrated that PGE2 and protein kinases are responsible for TRPV1 sensitization in vivo on a peripheral level. Even though we did observe abolishment of TRPV1 sensitizing factors upregulation as a result of FAAH inhibition, we were not able to observe any significant changes in *Ptgs2* mRNA levels.

A summary of all gene expression changes following pharmacological treatment with FAAH inhibitor and/or TRPV1 antagonist can be found in Table 1 and Table 2. However, it is noteworthy to mention that the observed effects were associated with acute administration of the dual acting compound, OMDM-198, while chronic OMDM-198 treatment could suppress inflammation through ECS and decrease the pain-sensitizing effectors of COX2 metabolism.

## 4. Materials and Methods

### 4.1. Animals

Male Wistar rats (Charles River, Hamburg, Germany) around 55th postnatal day, initially weighing 225–250 g, were used for all experiments. Animals were housed five per cage under a standard 12 h/12 h light/dark cycle with food and water available ad libitum. All experiments were approved by the Local Bioethics Committee of the Institute of Pharmacology (Cracow, Poland, approval number 938/2012 (date of approval: 28 June 2012).

### 4.2. Drugs and Reagents

**Figure 5:**
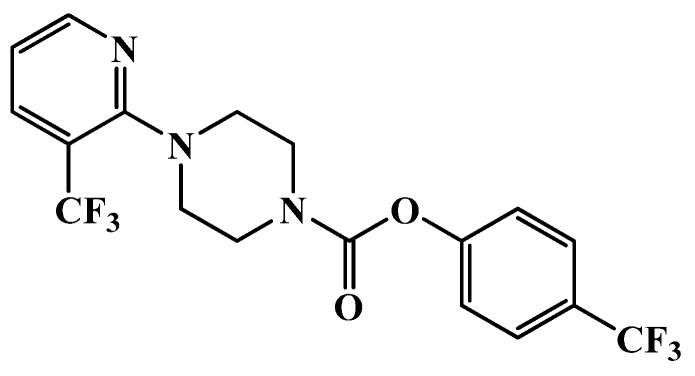
Chemical structure of OMDM198; the compound was synthesized on demand at the Department of Chemistry and Technology of Drugs in Sapienza University of Rome in Giorgio Ortar’s laboratory. All other compounds are commercially available.

URB597, SB366791 were obtained from Tocris Bioscience (Bristol, UK), whereas MIA, dimethyl sulfoxide (DMSO), Cremophor and Tween 80 were obtained from Sigma-Aldrich (Poznan, Poland). The OMDM-198 was synthesized, purified and characterized as described in Morera et al. 2009 [48] (Structure presented on Figure 5). All the reagents were dissolved in a vehicle solution. The vehicle for URB597 was 2% DMSO, 1% Tween 80, and 1% carboxymethyl cellulose in 0.9% saline. The vehicle for OMDM198 was 9% DMSO in 0.9% saline. The vehicle for SB366791 was 18% DMSO, 1% ethanol, and 1% Tween 80 in 0.9% saline.

### 4.3. OA Induction

Animals were deeply anaesthetized with 5% isoflurane in 100% O_2_ (4.5 L/min) until the flexor withdrawal reflex was abolished. The skin overlying the right knee joint was shaved and swabbed with 100% ethanol. A 27-gauge needle was introduced into the joint cavity through the patellar ligament and 3 mg of MIA, which is an irreversible NADPH inhibitor, diluted in 50 µL 0.9% saline was injected into the joint (intra-articular, i.a.) to induce OA-like lesions. MIA inhibits chondrocyte glycolysis and produces cartilage degeneration and subchondral bone alterations. The MIA model reproduces osteoarthritis-like histological lesions and functional impairment similar to that observed in human disease [49]. The rats were sacrificed at day 21 after MIA injection.

### 4.4. Treatment Paradigm

Rats were injected intraperitonealy (i.p.) with OMDM198 (the dual FAAH inhibitor and TRPV1 blocker) and respective single target compounds, SB366791 (TRPV1 antagonist) and URB597 (FAAH inhibitor) or vehicle, 1 h before decapitation at doses 1, 2, and 5 mg/kg, respectively. The vehicle group received a respective volume of 18% DMSO, 1% ethanol, and 1% Tween 80 in 0.9% saline. Moreover, a group of intact animals was used as a reference.

### 4.5. Tissue Isolation

After decapitation, L3–L5 dorsal lumbar spinal cord was collected from both the ipsilateral and contralateral side to the injury. Tissue samples were placed in individual tubes, frozen in liquid nitrogen, and stored at −80 °C until RNA and protein isolation.

### 4.6. RNA Preparation

Tissue was placed in 1 mL of TRIzol reagent (Invitrogen, Carlsbad, CA, USA). RNA isolation was performed according to Chomczynski’s method [50]. Tissue was homogenized in a tissue lyser (Qiagen Inc., Hilden, Germany). RNA was denaturated for 3 min at 70 °C. RNA concentration was measured using a NanoDrop ND-100 Spectrometer (Thermo Scientific, Wilmington, DE, USA). Each sample was equalized to a concentration of 1 μg/μL and reverse transcribed to cDNA using iScript Reverse Transcription Supermix (BioRad, Hercules, CA, USA) according to the manufacturer’s protocol in a 20 µL total volume. The complete reaction mix was incubated in a thermal cycler according to the manufacturer’s protocol.

### 4.7. Quantitative Polymerase Chain Reaction (qPCR)

The reaction was performed with TaqMan probes and TaqMan Universal PCR Super Mix (Bio-Rad, Hercules, CA, USA) in a thermocycler C1000™ CFX96™Real-Time system (Bio-Rad, Hercules, CA, USA) according to the manufacturer’s protocol: denaturation 30 s at 95 °C and then 40 cycles of: denaturation, 5 s at 95 °C, annealing, extension and plate read, 30 s at 60 °C. The expression of the hypoxanthine phosphoribosyltransferase 1 (*Hprt1*) transcript with a stable level between the control and investigated groups was quantified to control for variation in the cDNA amounts. As the quantity of PCR rises, the fluorescence coming from the probes used in the reaction rises proportionally. That allows us to monitor the amount of PCR product in real time between cycles. The threshold cycle (CT) value (cycle during which fluorescence exceeds the threshold value) for each gene was normalized with the CT value of the *Hprt1* reference gene. RNA abundance was calculated as 2^⁻Δ*C*t^. The results are presented as a fold change proportional to the expression level in intact animals. The following assays (TaqMan Gene Expression Assays, Life Technologies, Waltham, MA, USA) were used in the experiment: Rn01527840m1 (*Hprt1*), Rn02758689s1 (*Cb1*), Rn04342831s1 (*Cb2*), Rn00583117m1 (*Trpv1*), Rn00577086m1 (*Faah*), Rn00440861m1 (*Prkcg*), Rn01432300g1 (*Prkaca*), Rn00578842m1 (*Mapk14*), Rn00820922g1 (*Mapk3*), Rn01483828m1 (*Ptgs2*), Rn01461082_ml (*Alox12*). A total number of 10 animals per treatment group was used in the experiment.

### 4.8. Statistical Analysis

The analysis was performed using Prism V.5 (GraphPad Software, Inc., La Jolla, CA, USA). Changes in mRNA levels in the lumbar spinal cord were analysed using one-way analyses of variance. A Bonferroni post hoc test was used to compare the effects of MIA injection (vehicle-treated osteoarthritic group) to the intact group and pharmacological treatments for the vehicle-treated osteoarthritic group (ipsilateral or contralateral, respectively). The data were considered significant only when *p* < 0.05. * denotes significant differences vs. intact, whereas # denotes significant differences vs. vehicle treated osteoarthritic animals. **, ***, **** denote higher significance levels, whereas a horizontal line emphasizes significant differences between all treatments (including vehicle treated) groups vs. intact. Outlier values above/below mean ± 2 * standard deviation were excluded from the analysis.

## 5. Conclusions

Most pharmacological strategies to this date have been based on a specific single-target strategy that is now proving to be increasingly unsuccessful in treating diseases with a complex aetiology as in the case of OA. It has traditionally been classified as a noninflammatory arthritis; however, the dichotomy between inflammatory and degenerative arthritis is becoming less clear with the recognition of a plethora of ongoing immune processes. Actually, our results shed light on the inflammatory component of OA that is related to TRPV1 sensitization as presented by changes in *Trpv1* and mRNA kinase expression together with increases in *Alox12* expression. Apparent interplay between articular, immunological and the nervous systems in the pathophysiology of OA is a strong justification for novel multi-target drug development strategies. Our previous work had already presented the superior antinociceptive potential of OMDM198 over URB597, a FAAH inhibitor, and SB366791, a TRPV1 receptor antagonist. The experiments performed herein bring better understanding of the molecular underpinnings of OMDM198 with pronounced antinociceptive potential and prove its superiority over single-target treatment strategies.

## Figures and Tables

**Table 1:** Summary of observed pharmacological effects upon mRNA expression in the ipsilateral part of lumbar spinal cord. Data is presented as mean fold change ± SEM in comparison to intact animals. Values in brackets represent number of animals in given group. Values with *p* < 0.05 were considered significant. * Denotes significant differences vs. intact, whereas # denotes significant differences vs. the same side of the vehicle-treated osteoarthritic animals. **, ***, **** denote higher significance levels. ↑ denotes an increase vs. intact group, whereas a second ↑ denotes an increase vs. vehicle group. ↓ denotes a decrease vs. vehicle.

Ipsi	Vehicle	OMDM198	SB366791	URB597
*Cnr1*	4.36 ± 0.33 (10) ***↑	5.73934 ± 0.28 (10) ***^,#^↑↑	5.62706 ± 0.21 (10) ***^,#^↑↑	4.16213 ± 0.37 (7) ***
*Cnr2*	2.77949 ± 0.21 (9) **↑	2.829189 ± 0.4 (10) ***↑	1.76802 ± 0.14 (9)	2.166964 ± 0.29 (7)↑
*Trpv1*	1.75039 ± 0.11 (9) ***↑	1.86664 ± 0.15 (10) ***↑	1.78189 ± 0.12 (10) ***↑	1.80975 ± 0.15 (6) ***
*Faah*	3.67581 ± 0.35 (10) ***↑	3.91468 ± 0.43 (10) ***↑	3.08223 ± 0.17 (9) ***↑	2.63209 ± 0.36 (7) ***↑
*Mapk3*	2.847 ± 0.23 (10) ****↑	3.20392 ± 0.19 (10) ****↑	3.16103 ± 0.21(10) ****↑	3.30138 ± 0.36 (7) ****↑
*Mapk14*	3.38744 ± 0.46 (10) ****↑	2.56705 ± 0.08 (8)	3.01161 ± 0.1 (8) **↑	2.69419 ± 0.22 (6)↑
*Prkaca*	4.13981 ± 0.4 (10) ****↑	4.66819 ± 0.35 (6) ****↑	4.40495 ± 0.35 (6) ****↑	3.33948 ± 0.3 (7) ****
*Prkcg*	4.10269 ± 0.37 (10) ***↑	1.95838 ± 0.41 (9) ^#^↓	4.25047 ± 0.45 (6) ***↑	3.18495 ± 0.3 (7)↑
*alox12*	2.045551 ± 0.18 (10) *↑	1.519128 ± 0.11 (10)	1.876361 ± 0.2 (10)	1.866132 ± 0.18 (7)
*Ptgs2*	3.81417 ± 0.31 (10) ****↑	4.15244 ± 0.36 (10) ****↑	4.03079 ± 0.13 (10) ****↑	3.5141 ± 0.24 (7) ****↑

**Table 2:** Summary of observed pharmacological effects upon mRNA expression in the contralateral part of lumbar spinal cord. Data is presented as mean fold change ± SEM in comparison to intact animals. Values in brackets represent number of animals in given group. Values with *p* < 0.05 were considered significant. * Denotes significant differences vs. intact. **, ***, **** denote higher significance levels. ↑ denotes an increase vs. intact group.

Contra	Vehicle	OMDM198	SB366791	URB597
*Cnr1*	5.34396 ± 0.34 (8) ***↑	6.02753 ± 0.35 (10)***↑	6.20558 ± 0.19 (10) ***↑	5.0704 ± 0.34 (8) ***↑
*Cnr2*	4.075169 ± 0.49 (8) ***↑	2.604934 ± 0.1 (8) *↑	3.388387 ± 0.42 (10) ***↑	2.717181 ± 0.5 (9) **↑
*Trpv1*	1.98928 ± 0.12 (8) ***↑	1.8344 ± 0.11 (10) ***↑	1.85948 ± 0.07 (9) ***↑	1.9315 ± 0.19 (9) ***↑
*Faah*	4.63649 ± 0.49 (8) ***↑	3.49026 ± 0.27 (9) ***↑	4.23487 ± 0.28 (10) ***↑	3.38967 ± 0.55 (9) ***↑
*Mapk3*	3.69186 ± 0.28 (8) ****↑	3.52777 ± 0.22 (9) ****↑	3.72732 ± 0.19 (10) ****↑	3.98384 ± 0.54 (8) ****↑
*Mapk14*	3.87233 ± 0.45 (8) ****↑	3.02531 ± 0.29 (10) **↑	3.80339 ± 0.27 (10) ****↑	3.69974 ± 0.67 (9) ****↑
*Prkaca*	3.81766 ± 0.19 (7) ****↑	4.14995 ± 0.32 (8) ****↑	3.92648 ± 0.34 (10) ****↑	3.53792 ± 0.53 (9) ****↑
*Prkcg*	5.34829 ± 0.63 (8) ****↑	4.36753 ± 0.28 (9) ****↑	4.52286 ± 0.5 (10) ****↑	5.16141 ± 1.02 (9) ****↑
*alox12*	2.722801 ± 0.17 (8) ****↑	1.917924 ± 0.13 (9)	2.020144 ± 0.2 (10) *↑	2.324423 ± 0.3 (9) **↑
*Ptgs2*	4.27398 ± 0.32 (8) ****↑	3.89243 ± 0.2 (9) ****↑	4.05906 ± 0.14 (10) ****↑	3.86385 ± 0.46 (9) ****↑

## Abbreviations

2-AG	2-arachidonylglycerol
AEA	Anandamide
cAMP	Cyclic adenosine monophosphate
CB1	Cannabinoid receptor type 1
CB2	Cannabinoid receptor type 2
COX2	Cyclooxygenase-2
CT	Threshold cycle
DMSO	Dimethyl sulfoxide
DRGs	Dorsal root ganglions
ECS	Endocannabinoid system
FAAH	Fatty acid amide hydrolase
HETE	Hydroxyeicosatetraenoic acid
HPETE	Hydroperoxyeicosatetraenoic acid
MAPK	Mitogen-activated protein kinases
MIA	Monoiodoacetate
NSAIDs	Nonsteroidal anti-inflammatory drugs
OA	Osteoarthritis
qPCR	Quantitive polymerase chain reaction
PGE2	Prostaglandine E2
PGF2α	Prostamide F2α
PKA	Protein kinase A
PKC	Protein kinase C
TRPV1	Transient receptor potential cation channel subfamily V member 1
